# A confidence ellipse for the Net Reclassification Improvement

**DOI:** 10.1007/s10654-015-0001-1

**Published:** 2015-02-28

**Authors:** Kristin Mühlenbruch, Olga Kuxhaus, Michael J. Pencina, Heiner Boeing, Hannelore Liero, Matthias B. Schulze

**Affiliations:** 1 Department of Molecular Epidemiology, German Institute of Human Nutrition Potsdam-Rehbruecke, Arthur-Scheunert-Allee 114-116, 14558 Nuthetal, Germany; 2 German Center for Diabetes Research (DZD), Nuthetal, Germany; 3 Department of Biostatistics and Bioinformatics, Duke Clinical Research Institute, Duke University, Durham, NC USA; 4 Institute of Mathematics, University of Potsdam, Potsdam, Germany; 5 Department of Epidemiology, German Institute of Human Nutrition Potsdam-Rehbruecke, Nuthetal, Germany

**Keywords:** Risk assessment, Risk model, Model comparison, Reclassification, Confidence intervals

## Abstract

The Net Reclassification Improvement (NRI) has become a popular metric for evaluating improvement in disease prediction models through the past years. The concept is relatively straightforward but usage and interpretation has been different across studies. While no thresholds exist for evaluating the degree of improvement, many studies have relied solely on the significance of the NRI estimate. However, recent studies recommend that statistical testing with the NRI should be avoided. We propose using confidence ellipses around the estimated values of event and non-event NRIs which might provide the best measure of variability around the point estimates. Our developments are illustrated using practical examples from EPIC-Potsdam study.

## Background

Risk prediction models have become a main focus in epidemiological research in the past years. Although a large number of prediction models exists, of which some have already been integrated in treatment strategies or health promotion programs, there is an ongoing effort to improve prediction models by the use of new risk markers. For the evaluation of such model extensions, the Net Reclassification Improvement (NRI) was proposed by Pencina et al. in 2008 as an addition to the evaluation of discrimination, e.g. by comparing receiver operating characteristic curves [1]. The NRI is based on the calculation of the amount of correctly and incorrectly reclassified cases and non-cases comparing classification of individuals into a priori defined risk categories in terms of their predicted risk between two nested models. Since its publication it has been used in a growing number of studies, however, there is a large heterogeneity in its use, presentation, and interpretation [2, 3]. Especially with regard to testing statistical significance of NRI estimates, there remains uncertainty. Pencina [4] discussed that even small NRI values (<0.01) might produce statistically significant *p* values and Pepe et al. [5] points out that valid methods for inference for the NRI do not exist. In a recent review of NRI measures, Kerr et al. [2] raise concerns about the proposed test statistic and variance formula. This suggests that statistical testing should be avoided for the NRI measure. However, confidence intervals provide precision estimates and are preferable, not only for the overall NRI, but also for its components. The NRI components do not reflect an overall improvement but rather improvement among cases and non-cases separately. Therefore, our aim was to introduce a method to calculate a confidence ellipse around the two components of the NRI which reflects the precision of the estimates and can help interpret the magnitude and variability of the observed effects.

## Definition of NRI

Extension of prediction models with additional risk factors usually leads to changes in predicted risk for individual study participants. When predefined risk categories are used, this is reflected by upward and downward movements across these risk categories from the reference to the extended model. This reclassification is used for the calculation of the *NRI* which considers proportions of upward and downward movements separately for cases and non-cases (1) [1].

1 $$\begin{aligned} NRI_{cases} & = P\left( {up|case} \right) - P\left( {down|case} \right), \\ NRI_{{non{ - }cases}} & = P\left( {down|non{ - }case} \right) - P\left( {up|non{ - }case} \right), \\ NRI & = NRI_{cases} + NRI_{{non{ - }cases}} \\ & = P\left( {up|case} \right) - P\left( {down|case} \right) + P\left( {down|non{ - }case} \right) - P\left( {up|non{ - }case} \right) \\ & = \left[ {p_{up,cases} - p_{down,cases} } \right] + \left[ {p_{{down,non{ - }cases}} - p_{{up,non{ - }cases}} } \right] \\ \end{aligned}$$

The corresponding standard error for the *NRI* and its components was defined by Pencina et al. [4] and depends on the standard error of cases, which often is a much smaller group:

$$SE\left( {\widehat{NRI}} \right) = \sqrt {SE\left( {\widehat{NRI} _{cases} } \right)^{2} + SE\left( {\widehat{NRI} _{non - cases} } \right)^{2} } ,$$

$$SE\left( {\widehat{NRI}_{cases} } \right) = \sqrt {\frac{{\hat{p} _{up, cases} + \hat{p} _{down, cases} - \left( {\hat{p} _{up, cases} - \hat{p} _{down, cases} } \right)^{2} }}{{N_{cases} }} } ,$$

$$SE\left( {\widehat{NRI} _{non - cases} } \right) = \sqrt {\frac{{\hat{p} _{up, non - cases} + \hat{p} _{down, non - cases} - \left( {\hat{p} _{{up, non{ - }cases}} - \hat{p} _{down, non - cases} } \right)^{2} }}{{N_{non - cases} }}} .$$

As such, the *NRI* is the sum of the single components (*NRI*_*cases*_, *NRI*_*non*-*cases*_) reflecting improvement among cases or improvement among non-cases or both. Thereby, the overall measure does not include evaluation of improvement among cases or non-cases separately. Absolute risks are derived from regression models; either logistic regression or Cox-regression with the disease as the outcome variable.

## Confidence ellipse for two components of NRI

Pencina already suggested to report CIs for the *NRI* and used the bootstrap method for their construction [4]. Calculation of CIs would be informative not only for the overall NRI but also for the single components. Besides the bootstrapping method, CIs can be calculated with a formula related to the construction of CIs for independent proportions according to Agresti [6]; this approach will be applied further on. The standard errors for the overall *NRI* and its single components were defined before, so that the CIs can be defined as follows:

$$\left[ {\widehat{NRI} - z_{1 - \frac{\alpha}{ 2}} SE\left( {\widehat{NRI}} \right),\widehat{NRI} + z_{1 - \frac{\alpha}{ 2}} SE(\widehat{NRI})} \right]$$

with $z_{1 - \frac{\alpha}{ 2}}$ as the $\left( {1 - \frac{\alpha}{ 2}} \right)$-quantile of the standard normal distribution. The CIs for *NRI*_*cases*_ and *NRI*_*non*-*cases*_ can be calculated with the same method. While CIs of the two *NRI* components, *NRI*_*cases*_ and *NRI*_*non*-*cases*_, can be interpreted individually, this again would not allow an easy interpretation in terms of the overall improvement. To overcome this problem, we propose to use a confidence ellipse which allows evaluating the single components *NRI*_*cases*_ and *NRI*_*non*-*cases*_ in combination.

We introduce the following notation: Let $\theta = (\theta_{1, } \theta_{2} )$ be the parameter consisting of the *NRI* components, i.e.

$$\theta_{1} = NRI_{cases} = P\left( {up |case} \right) - P\left( {down |case} \right)\;{\text{and}}$$

$$\theta_{2} = NRI_{{non{ - }cases}} = P\left( {down |non{ - }case} \right) - P\left( {up |non{ - }case} \right)$$

We define the following probabilities

$$p_{1} = {\text{P}}\left( {up \cap case} \right),\, p_{2} = {\text{P}}\left( {down \cap case} \right),\, p_{3} = {\text{P}}\left( {up \cap non{ - }case} \right),\, p_{4} = {\text{P}}\left( {down \cap non{ - }case} \right)\;{\text{and}}\; p_{5} = {\text{P}}\left( {case} \right)$$

and can write $\theta$ as a function of these probabilities:

$$\theta = \left( {\theta_{1} ,\theta_{2} } \right) = g\left( \varvec{p} \right) = \left( {g_{1} \left( \varvec{p} \right),g_{2} \left( \varvec{p} \right)} \right) = \left( {\frac{{p_{1} - p_{2} }}{{p_{5} }}, \frac{{p_{4} - p_{3} }}{{1 - p_{5} }}} \right).$$

Consequently, the maximum likelihood estimates of $\theta_{1}$ and $\theta_{2}$ are given by the relative frequencies $\hat{p}_{j} = v_{j} /N$ (with $N = N_{cases} + N_{{non{ - }cases}}$) as follows:

$$\hat{\theta }_{1} = \frac{{\hat{p}_{1} - \hat{p}_{2} }}{{\hat{p}_{5} }} \;{\text{and}}\; \hat{\theta }_{2} = \frac{{\hat{p}_{4} - \hat{p}_{3} }}{{1 - \hat{p}_{5} }}$$

with

**Table:** 

	*Up*	*Down*	Total
*Case*	$v_{1}$	$v_{2}$	$v_{5}$
*Non*-*case*	$v_{3}$	$v_{4}$	$N - v_{5}$

Applying the multivariate central limit theorem to the vector of relative frequencies $\hat{\varvec{p}} = (\hat{p}_{1} ,\hat{p}_{2} , \hat{p}_{3} , \hat{p}_{4} , \hat{p}_{5} )^{T}$ we get, that for a large sample size $N$ the distribution of $\sqrt N (\hat{\varvec{p}} - \varvec{p})$ can be approximated by a five-dimensional normal distribution, i.e.,

2 $$\sqrt N \left( {\hat{\varvec{p}} - \varvec{p}} \right){ \mathop
\to \limits^{ D }} {\text{N}}_{5} \left( {0,A(\varvec{p})} \right).$$

Here $A(\varvec{p})$ is the covariance matrix of the limit distribution. It depends on the underlying probabilities $p_{j}$ and can be computed as:

$$A\left( \varvec{p} \right) = \left( {\begin{array}{*{20}l} {p_{1} (1 - p_{1} )} \hfill & { - p_{1} p_{2} } \hfill & { - p_{1} p_{3} } \hfill & { - p_{1} p_{4} } \hfill & {p_{1} (1 - p_{5} )} \hfill \\ { - p_{1} p_{2} } \hfill & {p_{2} (1 - p_{2} )} \hfill & { - p_{2} p_{3} } \hfill & { - p_{2} p_{4} } \hfill & {p_{2} (1 - p_{5} )} \hfill \\ { - p_{1} p_{3} } \hfill & { - p_{2} p_{3} } \hfill & {p_{3} (1 - p_{3} )} \hfill & { - p_{3} p_{4} } \hfill & { - p_{3} p_{5} } \hfill \\ { - p_{1} p_{4} } \hfill & { - p_{2} p_{4} } \hfill & { - p_{3} p_{4} } \hfill & {p_{4} (1 - p_{4} )} \hfill & { - p_{4} p_{5} } \hfill \\ {p_{1} (1 - p_{5} )} \hfill & {p_{2} (1 - p_{5} )} \hfill & { - p_{3} p_{5} } \hfill & { - p_{4} p_{5} } \hfill & {p_{5} (1 - p_{5} )} \hfill \\ \end{array} } \right).$$

With the help of the so-called delta method we can derive from (2) the asymptotic variance of $\hat{\theta } = (\hat{\theta }_{1} ,\hat{\theta }_{2} )$. Here we use, that $\hat{\theta } = g(\hat{\varvec{p}})$. To derive the asymptotic variance of $\hat{\theta }$ one has to multiply the matrix of partial derivatives of $g$ with $A(\varvec{p})$. This leads to

$$\sqrt N \left[ {\left( {\begin{array}{*{20}c} {\hat{\theta }_{1}
} \\ {\hat{\theta }_{2} } \\ \end{array} } \right) - \left(
{\begin{array}{*{20}c} {\theta_{1} } \\ {\theta_{2} } \\
\end{array} } \right)} \right] {\mathop \to \limits^{ D }}
{\text{N}}_{2} \left( {0,W(\varvec{p})} \right)$$

with $$W(\varvec{p}) = \left( {\begin{array}{*{20}c} {w_{1} } & 0 \\ 0 & {w_{2} } \\ \end{array} } \right)$$ and

$$w_{1} = \frac{{p_{1} + p_{2} }}{{p_{5}^{2} }} - \frac{{\left( {p_{1} - p_{2} } \right)^{2} }}{{p_{5}^{3} }}, w_{2} = \frac{{p_{3} + p_{4} }}{{(1 - p_{5} )^{2} }} - \frac{{\left( {p_{3} - p_{4} } \right)^{2} }}{{(1 - p_{5} )^{3} }}.$$

The asymptotic normality of $\hat{\theta }$ implies that

3 $$N\left( {\hat{\theta } - \theta } \right)^{T} W^{ - 1} \left(
{\hat{\varvec{p}}} \right)\left( {\hat{\theta } - \theta }
\right){\mathop \to \limits^{ D }} \chi_{2}^{2}$$

with $\chi_{2}^{2}$, the Chi squared distribution with two degrees of freedom and $W^{ - 1} \left( {\hat{\varvec{p}}} \right)$ is the inverse of the matrix $W(\varvec{p})$.

Because of the diagonal structure of $W(\varvec{p})$ and with asymptotic result from (3) we can define a $\left( {1 - \alpha } \right)$ confidence ellipse for $\theta$ as

$$\left\{ {\theta \in [ - 1,1]^{2} \mid \frac{{\left( {\hat{\theta }_{1} - \theta_{1} } \right)^{2} }}{{\hat{w}_{1} }} + \frac{{\left( {\hat{\theta }_{2} - \theta_{2} } \right)^{2} }}{{\hat{w}_{2} }} \le \frac{{\chi_{2;1 - \alpha }^{2} }}{N}} \right\}.$$

The determination of the confidence ellipse allows to determine the simultaneous precision of the NRI estimates for cases and non-cases.

Using previous notation and the following relationships $\hat{p}_{up, cases} = \hat{p}_{1} /\hat{p}_{5}$, $\hat{p}_{down, cases} = \hat{p}_{2} /\hat{p}_{5}$, $\hat{p}_{{up, non{ - }cases}} = \hat{p}_{3} /(1 - \hat{p}_{5})$ and $\hat{p}_{up, cases} = \hat{p}_{4} /(1 - \hat{p}_{5})$, the confidence ellipse can also be defined with the following equation.

$$\left\{ {\theta \in [ - 1,1]^{2} \mid \left( {\frac{{\widehat{NRI}_{cases} - \theta_{1} }}{{SE\left( {\widehat{NRI} _{cases} } \right)}}} \right)^{2} + \left( {\frac{{\widehat{NRI}_{{non{ - }cases}} - \theta_{2} }}{{SE\left( {\widehat{NRI} _{{non{ - }cases}} } \right)}}} \right)^{2} \le \chi_{2;1 - \alpha }^{2} } \right\}.$$

## Empirical data

### Study population

The European Prospective Investigation into Cancer and Nutrition (EPIC)-Potsdam study is a prospective cohort study initially including 27,548 participants aged 35–65 years. Details of recruitment and follow-up procedures were described previously [7, 8]. Briefly, within a median follow-up time of 7 years, 849 participants out of 25,167 participants free of diabetes at baseline developed incident diabetes. On this basis, the German diabetes risk score (GDRS) was developed using Cox-regression [9]. With the GDRS the 5-year risk for developing future type 2 diabetes can be calculated using information on lifestyle and anthropometric factors, diet and physical activity. It serves as the reference model in this underlying model comparison. We used data from 21,846 participants (727 cases) who had also information on family history of diabetes available. The extended model additionally included family history; this model was compared with the reference model. Table 1 shows the reclassification of cases and non-cases due to model extension based on the use of 5 predefined risk categories.

**Table 1:** Reclassification table by cases and non-cases resulting from adding family history of diabetes to the German DRS (GDRS), EPIC-Potsdam cohort (N = 21,846)

*N* (%)	GDRS + family history					Total
	1: Low	2: Still low	3: Increased	4: High	5: Very high	
*Cases*						
1: Low^a^	*21 (2.89)*	7 (0.96)	–	–	–	28 (3.85)
2. Still low	13 (1.79)	*102 (14.03)*	30 (4.13)	–	–	145 (19.94)
3. Increased	–	32 (4.40)	*176 (24.21)*	61 (8.39)	–	269 (37.00)
4. High	–	–	29 (3.99)	*146 (20.08)*	36 (4.95)	211 (29.02)
5. Very high	–	–	–	15 (2.06)	*59 (8.12)*	74 (10.18)
Total	34 (4.68)	141 (19.39)	235 (32.32)	222 (30.54)	95 (13.07)	727 (100)
*Non*-*cases*						
1. Low	*9001 (42.62)*	625 (2.96)	–	–	–	9626 (45.58)
2. Still low	1415 (6.70)	*4220 (19.98)*	672 (3.18)	–	–	6307 (29.86)
3. Increased	–	858 (4.06)	*2613 (12.37)*	387 (1.83)	–	3858 (18.27)
4. High	–	–	269 (1.27)	*782 (3.70)*	98 (0.46)	1149 (5.44)
5. Very high	–	–	–	40 (0.19)	*139 (0.66)*	179 (0.85)
Total	10,416 (49.32)	5703 (27.0)	3554 (16.83)	1209 (5.72)	237 (1.12)	21,119 (100)

^a^Risk categories were created according to score points of the German Diabetes Risk Score: low risk: <410 points (5-year risk < 0.88 %); still low: 410–<510 (0.88–<2.37 %); increased risk: 510–<610 (2.37–<6.30 %); high risk: 610–<710 (6.30–<16.21 %); very high risk: ≥710 (≥16.21 %)

*NRI* measures were calculated as follows

$$NRI_{cases} = (\left( {0.96 + 4.13 + 8.39 + 4.95} \right) - \left( {1.79 + 4.40 + 3.99 + 2.06} \right))/100 = (18.43 - 12.24)/100 = 0.0619$$

$$NRI_{{non{ - }cases}} = (\left( {6.70 + 4.06 + 1.27 + 0.19} \right) - \left( {2.96 + 3.18 + 1.83 + 0.46} \right))/100 = (12.22 - 8.43)/100 = 0.0379$$

$$NRI = 0.0619 + 0.0379 = 0.0998$$

### Calculation of Confidence Intervals and Confidence ellipses

**Fig. 1:**
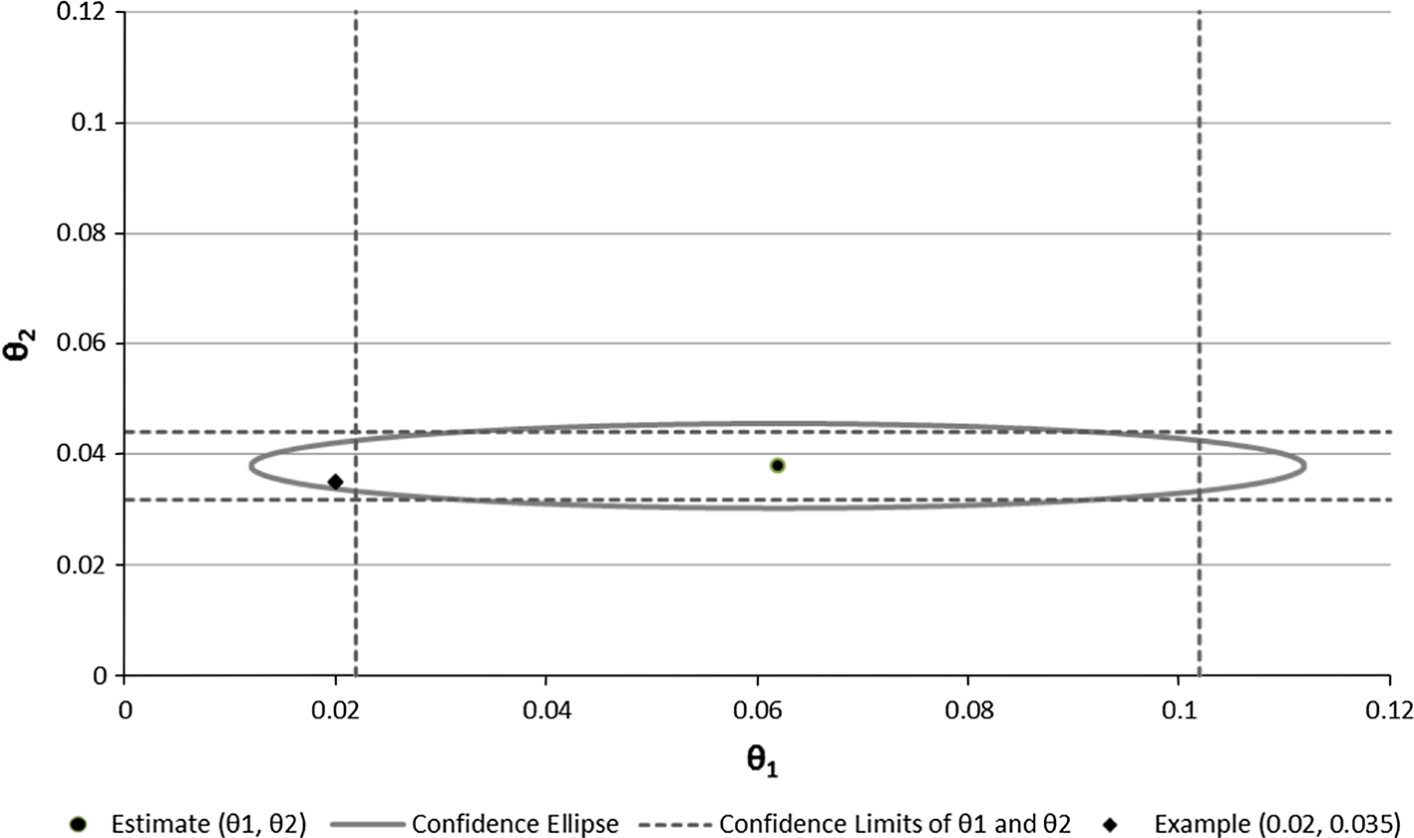
Confidence ellipse for the two-dimensional estimate *θ * = (*θ*_1_, *θ*_2_) of the calculated *NRI*_*cases*_ and *NRI*_*non-cases*_, EPIC-Potsdam study. *NRI* for cases and *NRI* for non-cases were calculated by comparing the German diabetes risk score extended with family history of diabetes with the initial German diabetes risk score; five predefined risk categories were used for calculation; the confidence ellipse was defined with the accepted *θ*_*1*_ and *θ*_*2*_ values for *α* = 0.05. The area within the ellipse defines the area of accepting the null hypotheses $H_{0}: NRI_{cases} = \theta_{1}$ *and* $NRI_{{non{ - }cases}} = \theta_{2}$ and the area outside the ellipse is the area of not accepting $H_{0}$. The *black dot* is the estimated *θ* for *NRI*_*cases*_ and *NRI*_*non*-*cases*_. The *horizontal* and *vertical reference lines* display the lower and upper confidence limits of *NRI*_*non*-*cases*_ (0.0318, 0.0440) and of *NRI*_*cases*_ (0.0219, 0.1019) respectively. The *black diamond* displays the discussed example for null hypotheses: *NRI*_*cases*_ = 0.02 *and* *NRI*_*non*-*cases*_ = 0.035

Based on the asymptotic method we determined 95 % CIs for *NRI*_*cases*_ and *NRI*_*non*-*cases*_ (Fig. 1). Taking into account the large number of non-cases it is obvious that estimation of *NRI*_*non*-*cases*_ was much more precise than of *NRI*_*cases*_. The calculation of CIs for single components does not allow evaluating both components in combination.

Therefore, we computed a confidence ellipse for *NRI*_*cases*_ and *NRI*_*non*-*cases*_ to reflect precision of their estimates in combination and which also allows to evaluate the area of acceptable values. Figure 1 shows CIs for the single components (vertical and horizontal lines) as well as the confidence ellipse, both approaches were based on the five risk categories described before. When constructing CIs for the components separately, *NRI*_*cases*_ (0.0619) has a CI of 0.0219–0.1019. Therefore, the value 0.02 lies outside of this interval while the *NRI*_*non*-*cases*_ (0.0379) had a CI ranging from 0.0318 to 0.0440 thus including a value of 0.035. Using both CIs separately would therefore lead to the conclusion that *NRI*_*cases*_ is significantly higher than 0.02 while *NRI*_*non*-*cases*_ is not significantly higher than 0.035. However, examining the vector (0.02, 0.035) within the confidence ellipse we can see that it is located inside the area of the ellipse. Thus, the confidence ellipse indicates that—when evaluated together—neither is the *NRI*_*cases*_ different from 0.02 nor is the *NRI*_*non*-*cases*_ different from 0.035. This example clearly indicates that evaluating single *NRI* components separately might result in different decisions than evaluating the single components in combination by the use of confidence ellipses.

These results were based on the asymptotic method for both the calculation of CIs and of the confidence ellipse.

## Discussion

The use of the *NRI* is informative for the evaluation of improvements of prediction models when taking into account the obvious limitations associated with the use of categories and cut-offs. Given that no established cut-offs for the *NRI* exist which allow interpreting its value as being meaningful from a clinical or public health point of view, reliance solely on significance testing has been frequently adopted in reclassification analyses.

As recommended in a recent review of the NRI methods [3], it is preferable to investigate model improvement separately for cases or non-cases. A general framework for testing the two components of the overall *NRI*, *NRI*_*cases*_ and *NRI*_*non*-*cases*_, has previously been laid out by Pencina et al. [1]. However, a major drawback of examining single components in isolation is that the results cannot be interpreted in terms of the overall model improvement. We note that recent recommendations suggest not applying statistical testing at all [2, 3]. Likewise, our developments facilitate the use of confidence intervals. A particularly appealing approach is based on using the confidence ellipse which reflects the 2-dimensional nature of the situation. Our empirical example indicates that confidence ellipses can be useful in reflecting both, the precision of the *NRI* estimation as well as putting the results in the context of overall improvement.

Our proposed method of confidence ellipses is also flexible here as it can be applied to evaluating extensions of prediction models using equal or different weights as well as thresholds of acceptable model improvement for cases and non-cases as already discussed by Greenland [10].

In conclusion, confidence ellipses might be particularly useful in the context of evaluating overall or case- versus non-case-specific model improvement as they allow evaluating varying acceptable values of the *NRI* components in combination and also reflect the precision of their estimates.
